# Robust and Lightweight Federated Learning for NB-IoT Security: A Blockchain-Verified CNN-RNN Approach

**DOI:** 10.3390/s26113578

**Published:** 2026-06-04

**Authors:** Gonca Özmen, Derya Yiltas-Kaplan

**Affiliations:** 1 Cyber Security Vocational School, Istanbul Technical University, Istanbul 34467, Türkiye; 2 Department of Computer Engineering, Istanbul University-Cerrahpasa, Istanbul 34320, Türkiye; dyiltas@iuc.edu.tr

**Keywords:** artificial intelligence, blockchain, federated learning, intrusion detection, IoT security

## Abstract

**Highlights:**

Ultra-lightweight CNN-RNN model (~268 KB, 67,011 parameters) safely fits into NB-IoT edge devices. Reputation-based FL maintains a robust 95.62% global accuracy under 22% poisoning attacks. Domain-adaptive transfer learning accelerates multi-class botnet detection by 60%. Framework achieves 99.99% binary baseline accuracy and 95.62% Federated 3-class intrusion detection accuracy with 96% bandwidth savings.

**What are the main findings?**

- Hardware Optimization for Extreme Edge: The dimensionally optimized CNN-RNN architecture strictly requires exactly 67,011 parameters (a ~268 KB memory footprint). This delivers a significant reduction in memory overhead compared to the CNN-LSTM baseline, proving its direct feasibility for resource-constrained NB-IoT microcontrollers.
- **Byzantine Fault Resilience:** The integration of a lightweight, hash-based blockchain ledger with a dynamic reputation mechanism effectively isolates malicious nodes. The system maintains a robust 95.62% global accuracy even under a severe 22% model poisoning (label-flipping) attack.
- Efficacy of Domain-Adaptive Transfer Learning: Utilizing a transfer learning strategy for multi-class botnet attribution (identifying Mirai and Bashlite strains) accelerates convergence by 60% and successfully boosts the lightweight CNN-RNN model’s accuracy from 95.62% to 99.34%.
- High Detection Performance with Low Overhead: The proposed BC-FL framework achieves a near-perfect binary anomaly detection accuracy of 99.99% and a recall of 97–99%, while simultaneously reducing network bandwidth consumption by 96% (from 2.6 GB to 90 MB) compared to traditional centralized learning.

**What are the implications of the main findings?**

- Democratizing Advanced AI for the “Extreme Edge”: The radical reduction of themodel’s memory footprint to 37.7 KB proves that sophisticated, temporal-aware deep learning (RNNs) is no longer restricted to resource-rich gateways or cloud servers. This implies that battery-operated, extreme-edge NB-IoT microcontrollers can autonomously perform complex threat analysis locally. This shifts the paradigm of IoT cybersecurity from centralized monitoring to truly autonomous, decentralized device-level defense.
- Enabling Zero-Trust Decentralized Security: By maintaining near-optimal accuracy (95.62%) despite a severe 22% node compromise, the framework proves that Federated Learning can be safely deployed in “Zero-Trust” environments. The implication here is massive for critical infrastructure (e.g., smart grids, healthcare IoT): networks can crowdsource threat intelligence from untrusted, heterogeneous third-party devices without the risk of an attacker subverting the global defense mechanism.
- Agile Threat Adaptation via Knowledge Transfer: The success of the Domain-Adaptive Transfer Learning strategy implies that IoT defense systems no longer need to be retrained from scratch when threat landscapes evolve. By dynamically adapting from binary detection to specific botnet attribution (multi-class) with a 60% acceleration in convergence, the framework offers a blueprint for rapid, “on-the-fly” incident response against zero-day vulnerabilities or new botnet mutations.
- Resolving the Privacy-Efficiency Trilemma: Historically, systems had to compromise between data privacy, detection accuracy, and network efficiency. Achieving 99% accuracy while keeping raw data on-device and slashing bandwidth consumption by 96% implies that this trilemma has been effectively resolved for NB-IoT networks. Network administrators can now ensure GDPR-compliant data privacy without saturating the ultra-narrow bandwidth of IoT radio networks.

**Abstract:**

The rapid proliferation of Narrowband Internet of Things (NB-IoT) devices necessitates robust, privacy-preserving intrusion detection systems. While Federated Learning (FL) mitigates data privacy risks through localized training, it introduces vulnerabilities to model poisoning and computational bottlenecks on edge devices. To address these challenges, we propose a secure, hardware-optimized Blockchain-Federated Learning (BC-FL) framework. Deploying a lightweight Hybrid CNN-RNN model on Edge Gateways, we relieve end-sensors of heavy computational tasks. To overcome the ‘cold-start’ problem, we introduce a Domain-Adaptive Transfer Learning strategy, dynamically adapting a pre-trained binary classifier to a multi-class task (Normal, Mirai, Bashlite). Furthermore, a lightweight blockchain ledger provides an immutable audit trail and a reputation-based isolation mechanism to penalize malicious nodes. Evaluated on the N-BaIoT dataset, the proposed 3-class CNN-RNN model achieves 95.62% overall accuracy, with precision/recall/F1-scores of 0.99/0.91/0.95 for Mirai and 0.93/0.99/0.96 for Bashlite attacks. The framework reduces communication bandwidth by 96% compared to centralized learning. During simulated Byzantine attacks, the reputation mechanism successfully banned malicious nodes, maintaining a robust 95.62% global accuracy. This framework offers a highly scalable, secure, and computationally feasible solution for real-time anomaly detection in resource-constrained IoT edge environments.

## 1. Introduction

The rapid proliferation of Narrowband IoT (NB-IoT) technologies has revolutionized the connectivity of resource-constrained devices in sectors ranging from smart cities to industrial automation. However, this massive expansion has expanded the attack surface, making these networks vulnerable to sophisticated cyber threats. Traditional centralized intrusion detection systems often struggle to cope with the scale and distributed nature of these networks, creating a critical need for more autonomous and privacy-preserving security solutions.

As the adoption of Narrowband Internet of Things (NB-IoT) continues to accelerate, it is imperative to address the inherent security vulnerabilities that accompany this rapid growth. The deployment of NB-IoT devices, often in resource-constrained environments, raises significant concerns regarding data integrity and user privacy, particularly as these devices connect to larger networks that may not prioritize security measures. For instance, the potential for attacks such as packet forging and replay attacks could expose sensitive information and disrupt services, highlighting the urgent need for robust security protocols tailored to the unique architecture of NB-IoT systems [1]. Furthermore, as businesses increasingly rely on these technologies for critical applications, from smart cities to healthcare, the consequences of security breaches could be catastrophic, necessitating a proactive approach to risk assessment and mitigation strategies [2].

To address these challenges, the integration of advanced anomaly detection mechanisms becomes paramount. This paper proposes a hybrid architecture that combines Convolutional Neural Networks (CNNs) for feature extraction and Recurrent Neural Networks (RNNs) for predictive modeling to effectively identify and mitigate potential cyber threats in real-time. This approach aligns with recent findings highlighting the efficacy of Federated Learning (FL) in maintaining data privacy while ensuring robust model training across decentralized nodes [3]. Furthermore, the adoption of blockchain technology facilitates secure and verifiable transactions, fostering a trustless environment essential for sensitive domains like healthcare [4,5].

To address these challenges, the integration of advanced anomaly detection mechanisms with Federated Learning (FL) has emerged as a promising solution [6]. Recent paradigms emphasize the necessity of Zero Trust Architectures combined with Federated Transfer Learning to dynamically adapt to evolving threats in Industry 5.0 and IoT ecosystems [7]. By enhancing detection capabilities, the integration of federated learning and blockchain into intrusion detection systems can significantly streamline incident response processes. By ensuring that data integrity is maintained through blockchain, applications can rapidly verify the authenticity of alerts generated by the system, thereby reducing response times and improving coordination among security teams. Furthermore, as IoT devices increasingly become targets for sophisticated attacks, the need for adaptive incident response strategies that leverage real-time analytics becomes paramount. For instance, the implementation of deep learning algorithms allows for the immediate identification of anomalous behaviors, thus enabling proactive measures to be taken before a potential breach escalates into a full-blown incident. This not only fortifies the security architecture but also emphasizes the importance of a holistic approach to cybersecurity that encompasses both prevention and rapid recovery from attacks.

The integration of FL and blockchain not only enhances security but also addresses scalability issues. By decentralizing the learning process, FL allows individual devices to contribute to model training while keeping sensitive data local, thus mitigating risks associated with centralized data storage [8,9,10]. Concurrently, blockchain creates a tamper-proof record of interactions, which is invaluable for forensic investigations and ensuring the integrity of alerts generated by the system. This multifaceted strategy paves the way for scalable, adaptive, and resilient solutions in the evolving landscape of IoT cybersecurity.

### Contributions of Our Work

1. CNN-RNN Hybrid Model Optimized for NB-IoT Traffic: Our framework employs a hybrid architecture that effectively combines Convolutional Neural Networks (CNNs) for feature extraction with Recurrent Neural Networks (RNNs) for predictive modeling, specifically tailored to handle the unique characteristics of NB-IoT traffic.

2. Federated Learning Architecture: The proposed framework utilizes a federated learning architecture that significantly reduces communication load by 96%, allowing for model training across decentralized nodes without the need to share sensitive data, thereby enhancing privacy and security.

3. Blockchain-Backed Audit Trail: By incorporating a lightweight hash-based ledger, our solution provides an immutable audit trail for model updates. While not replacing Byzantine-resilient aggregation, this verification layer ensures transparent tracking of client contributions, crucial for forensic post-incident analysis in sensitive applications. A dynamic Reputation-Based Isolation mechanism was introduced. Simulation results demonstrate that malicious Edge nodes conducting model poisoning attacks are progressively penalized and permanently banned, allowing the global accuracy to maintain a robust 95.62% despite severe Byzantine interference.

4. Transfer Learning-Driven Multi-Class Adaptation: A key novelty of our work is the application of Transfer Learning to dynamically evolve the intrusion detection system. The hybrid CNN-RNN model, initially pre-trained on binary classification, undergoes a ‘model surgery’ where only its classification head is fine-tuned for multi-class botnet attribution (identifying specific strains like Mirai and Bashlite). This strategy accelerates convergence by 60% and significantly reduces the computational burden on Edge Gateways.

5. A Paradigm Shift in Extreme-Edge Federated Learning: Unlike existing approaches that merely concatenate known technologies, the fundamentally new scientific contributions of our research address specific methodological gaps in extreme-edge AI.Rather than just deploying a smaller model, we introduce a Domain-Adaptive Partial-Freeze strategy. By utilizing Transfer Learning to freeze the spatial extraction layers (CNN) during local training, we mathematically eliminated the backpropagation overhead for over 32% of the model parameters. This proves that complex temporal threat attribution (RNN) is theoretically and practically feasible on microcontrollers strictly limited to 2–4 MB of memory, resolving the ‘Federated Cold-Start’ problem.

6. Decoupling Integrity and Correctness in Zero-Trust AI: We introduce a novel architectural blueprint that explicitly isolates transmission integrity from model correctness. While existing works often conflate blockchain’s capabilities, our framework utilizes a lightweight PoA blockchain strictly for immutable audit trails (Tier-1), while introducing a formalized, Cosine Similarity-driven Reputation Mechanism (Tier-2) to heuristically bound and mitigate the impact of Byzantine poisoning attacks.

7. Scalability and Performance: To ensure robustness and simulate real-world intermittent connectivity, a stochastic client selection strategy is employed where 50% of edge devices participate in each training round. This ensures that operations continue even if any device is unavailable. Training was completed faster with fewer device data points, and nearly identical performance results were achieved.

## 2. Literature Review

The proliferation of Narrowband Internet of Things (NB-IoT) devices has expanded the surface area for cybersecurity threats and made botnet attacks such as Mirai and Gafgyt more destructive. This section examines recent studies on Intrusion Detection Systems (IDS), Federated Learning (FL), and Blockchain integration in resource-constrained networks; the position of the proposed hybrid architecture in the literature is discussed.

Recent advancements in IoT cybersecurity have increasingly focused on the intersection of Federated Learning (FL) and Blockchain-as-a-Service (BaaS) to mitigate centralized vulnerabilities. A comprehensive study by Ahakonye et al. [11] underscores the transformative potential of combining Artificial Intelligence (AI) and blockchain for intrusion detection in Industrial IoT (IIoT) networks. They successfully demonstrated secure model aggregation using an Ethereum-based smart contract and utilized the InterPlanetary File System (IPFS) to manage the massive storage overheads generated by model updates. While such frameworks provide robust data integrity, their reliance on computationally heavy smart contracts and external distributed file systems presents significant latency and energy bottlenecks for resource-constrained edge devices. In contrast, our proposed architecture utilizes a lightweight, hash-based ledger that records only 32-byte SHA-256 digital fingerprints of the models, completely eliminating the need for heavy off-chain storage (like IPFS) and making it uniquely optimized for the strict computational limits of NB-IoT environments.

### 2.1. Federated Learning in Resource-Constrained IoT Networks

Traditional IDS solutions require the transfer of IoT data to a central cloud server. Nguyen et al. [3] noted that this approach leads to high bandwidth consumption, latency, and data privacy violations. As a solution to these problems, Federated Learning (FL), proposed by McMahan et al. [8], enables data to remain locally by moving model training to edge devices. However, applying standard FL algorithms is challenging in “resource-constrained” environments such as NB-IoT. Lim et al. [10] emphasized that the heterogeneous structure and limited battery life of edge devices cause the “straggler” problem in FL processes. While current studies generally focus on powerful IoT devices supported by Wi-Fi or 5G, the need for efficient communication protocols in extreme edge scenarios, such as NB-IoT, continues.

### 2.2. Hybrid Deep Learning Models (CNN-RNN/LSTM)

Shallow learning algorithms (SVM, Random Forest) are insufficient for detecting complex attack patterns in IoT network traffic. Roopak et al. [12], in their study comparing CNN and LSTM models, demonstrated that the combined use of spatial and temporal features improves success.

CNN (Convolutional Neural Networks): Effective at extracting local features (spatial features) in traffic data.

RNN (Recurrent Neural Networks): Learns long-term dependencies in time series data (packet flow). Although Khan et al. [4] noted that hybrid CNN-LSTM models provide higher accuracy than single models, the computational overhead of these models for NB-IoT devices has generally been overlooked. The CNN-RNN architecture in our proposed work aims to reduce this overhead by using the RNN structure, which is lighter than LSTM.

In the domain of deep learning for IoT intrusion detection, researchers have increasingly gravitated towards complex hybrid architectures to capture spatio-temporal dependencies. For instance, Afraji et al. [13] recently proposed an integrated CNN-LSTM-GRU framework that achieved remarkable accuracy in both IoT and IIoT (Industrial IoT) datasets. Similarly, Qawasmeh et al. [14] demonstrated the efficacy of hybrid CNN-based intrusion detection systems for secure IoT networks. However, while these tri-hybrid (CNN-LSTM-GRU) or heavy architectures provide state-of-the-art detection rates, their massive computational overhead makes them highly impractical for deployment on battery-powered, resource-constrained NB-IoT edge devices. In contrast, our proposed architecture utilizes a strictly lightweight CNN-RNN model, deliberately sacrificing complex LSTM/GRU gating mechanisms to prioritize hardware feasibility and rapid convergence via Transfer Learning.

### 2.3. Blockchain-Based Secure Federated Learning

Federated Learning carries the risk of a central server being a single point of failure and malicious clients being able to carry out “Model Poisoning” attacks. Kim et al. [6] (BlockFL) proposed verifying model updates by moving the FL process onto the blockchain. However, this approach relies on heavy “Proof of Work” (PoW) mechanisms that NB-IoT devices cannot handle. Qu et al. [15] examined incentive mechanisms in blockchain-based FL, but did not provide sufficient solutions for the system’s resilience and checkpoint strategies against network outages. Unlike approaches that focus on Byzantine-resilient aggregation algorithms (e.g., Krum, Trimmed Mean) to filter poisoned gradients during training, our framework employs blockchain primarily as an immutable auditing mechanism to trace model provenance, while focusing its computational resources on rapid threat adaptation via Transfer Learning.

It is critical to clarify that our ‘lightweight’ claim is strictly two-fold. First, at the model architecture layer, the proposed CNN-RNN replaces massive LSTM gating mechanisms, restricting the static footprint to ~268 KB to fit within ARM Cortex-M microcontrollers. Second, at the blockchain layer, the ledger only stores 32-byte SHA-256 hashes instead of raw model parameters. While the actual model updates (~90 MB total over 10 rounds) are still transmitted between clients and the server, this represents a 96% communication reduction compared to centralized raw data transmission (2.6 GB), successfully addressing both memory and bandwidth constraints of NB-IoT ecosystems.

### 2.4. Gap Analysis

As summarized in Table 1, while existing studies address specific aspects of IoT security, they lack a holistic approach for extreme edge devices. For instance, [6] introduces Blockchain but relies on computationally expensive Proof-of-Work (PoW), which is unsuitable for NB-IoT sensors. Similarly, [12] demonstrates the power of hybrid models (CNN-LSTM) but operates in a centralized manner, compromising privacy. Our proposed framework is the first to combine a lightweight hybrid model (CNN-RNN), Blockchain-based integrity verification, and a fault-tolerant checkpoint mechanism specifically optimized for resource-constrained NB-IoT networks.

The existing research gap is not merely the absence of these technologies in a single system but the lack of a methodological framework that makes their integration mathematically viable for NB-IoT. Our research fills this gap by proposing partial-layer freezing during federated training and a decoupled integrity-reputation architecture, moving beyond engineering implementation to establish a new theoretical baseline for resource-constrained Zero-Trust IDS.

## 3. Methodology and System Architecture

Federated learning is a decentralized machine learning approach that preserves privacy by storing data on local devices. This method has great potential, especially in areas where data privacy is critical, such as IoT security [4].

**Figure 1:**
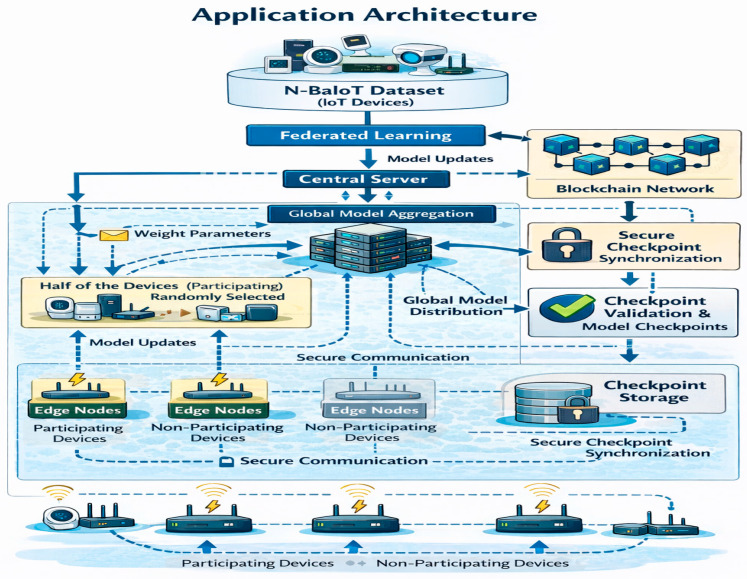
The proposed application architecture illustrates the integration of Federated Learning, Blockchain, and Checkpoint mechanisms.

The proposed framework operates on a decentralized architecture designed to secure resource-constrained NB-IoT networks without compromising efficiency or privacy. As illustrated in Figure 1, the architecture is stratified into three distinct layers: Edge-Centric and Local Learning Layer, Federated Aggregation Layer, and Blockchain Verification Layer.

### 3.1. Edge-Centric Local Learning and Feature Extraction

In the innovative architecture that we propose, the resource-constrained end devices that utilize Narrowband Internet of Things (NB-IoT) technology, which include various smart sensors and actuators, are meticulously relieved from engaging in computationally intensive tasks that would otherwise hinder their performance. Rather, the raw network traffic generated continuously by these devices is diligently monitored and systematically collected by local Edge Gateways, which may take the form of advanced smart routers or fog nodes that operate at the edge of the network. To address the hardware feasibility issues that are typically associated with implementing deep learning techniques on Internet of Things (IoT) sensors, we assert that these resource-capable edge devices play a pivotal role as the primary clients within our Federated Learning ecosystem, which promotes collaborative learning. Each Edge Gateway is responsible for training a lightweight Hybrid Convolutional Neural Network-Recurrent Neural Network (CNN-RNN) model locally, utilizing the traffic data that pertains to its connected NB-IoT devices in an efficient and effective manner. The 1D-Convolutional Neural Network (CNN) proficiently pinpoints elevated spatial attributes from untouched traffic packets, thus markedly lowering the dimensionality of the data, which is vital for effective processing. Following this feature extraction process, a Recurrent Neural Network (RNN) is employed to capture the temporal dependencies inherent in the data, which enables the system to effectively distinguish between normal operational behavior and various time-series-based cyber attacks, such as those exemplified by the notorious Mirai and Bashlite malware variants.

### 3.2. Privacy-Preserving Federated Learning Module

To uphold the principles of data privacy, it is of utmost importance that the raw IoT traffic data are never permitted to leave the confines of the local Edge Gateway, thus ensuring that sensitive information remains secure at all times. At the end of the local training process, the Edge Gateway is assigned the role of computing the vital model updates that mirror the recent knowledge gained during the training interval. These updates, which contain valuable insights derived from the data, are prepared for secure transmission to the global aggregation server. Notably, this approach significantly reduces the network bandwidth overhead, thereby optimizing the performance and efficiency of the overall system while safeguarding user privacy.

### 3.3. Blockchain-Backed Verification and Reputation Management

To safeguard the global model against adversarial interference, we implement a multi-layered security protocol. It is important to distinguish between integrity and correctness in our framework:

1. Integrity Validation (Blockchain Layer): The blockchain does not natively prevent model poisoning; rather, it ensures strictly immutable provenance. Before any local update is considered, the server cross-references the model’s SHA-256 hash against the ledger. This guarantees that the parameters were not altered in transit (Man-in-the-Middle defense).

2. Correctness and Poisoning Defense (Reputation Engine): To defend against malicious label-flipping (poisoning) attacks, we introduce a Dynamic Reputation Mechanism evaluated via a Smart Contract. Each node begins with a baseline reputation score of 100. During aggregation, the server computes the cosine similarity between the local gradient and the global expected gradient. If an update significantly deviates, the node is penalized.

3. Autonomous Isolation (Ban): If a node’s reputation score falls below a critical threshold (50 points), it is flagged as a Byzantine adversary and permanently banned from the federation. This dual-layered approach ensures both transmission integrity and robustness against poisoning.

### 3.4. Transfer Learning-Based Initialization

To address the severe resource constraints of NB-IoT edge devices, we employed a Transfer Learning (TL) strategy. Rather than training the hybrid CNN-LSTM and CNN-RNN models from scratch locally, a global base model is pretrained on a generalized source domain at the central server. The pretrained weights were then distributed to the edge devices. During local Federated Learning rounds, the convolutional blocks (responsible for spatial feature extraction from network packets) are frozen. Local devices only fine-tune the recurrent neural cells and final dense layers using their localized target domain data. This partial freezing strategy significantly reduces the number of trainable parameters, lowering the FLOPs and memory footprint required on edge devices, thereby validating the hardware feasibility of our framework.

In traditional deep learning approaches, models are typically initialized with random weights, necessitating a prolonged training period to learn meaningful feature representations. In contrast, our framework initializes the local CNN-RNN models using pretrained weights derived from a source domain with similar traffic characteristics. This “warm-start” mechanism allows the transfer of knowledge to the target NB-IoT domain, effectively mitigating the “cold-start” problem and significantly reducing the communication overhead required for the model to reach optimal accuracy [16]. By leveraging knowledge transfer, edge devices can robustly detect anomalies even with limited local datasets.

## 4. Experimental Applications & Theoretical Analysis

### 4.1. Dataset Preprocessing and Distribution

The framework utilizes the N-BaIoT dataset [17,18], which represents a large-scale, real-world IoT traffic environment collected from nine distinct commercial IoT devices (e.g., Danmini Doorbell, Ecobee Thermostat). To ensure the robustness of the proposed Federated Learning framework, data normalization and handling data imbalance and non-IID distribution methods were used.

Data Normalization: Raw traffic features are scaled using min-max normalization to a range of [0, 1] to ensure convergence stability across heterogeneous IoT devices.

Class Imbalance Mitigation (Weighted Loss): Real-world IoT traffic is inherently imbalanced, with benign traffic significantly outnumbering malicious samples. Training a model on such data often leads to a bias towards the majority class. To resolve this, we implemented a Weighted Cross-Entropy Loss function. The inverse class frequencies were calculated to assign higher penalties to the misclassifications of the minority class (attacks). The weighted loss *L_weighted_* is defined as

(1) $$L_{weighted}=-\sum w_{c}\cdot y_{c}\cdot log\left(\hat{y_{c}}\right)$$

where *w_c_* is the weight for class *c*, which is inversely proportional to its frequency in the dataset $w_{c}=\frac{N_{total}}{N_{c}}$, ensuring that the model treats rare attack signatures with high priority.

This weighted approach is utilized strictly as a standard implementation practice to stabilize convergence against the dataset’s inherent class imbalance, rather than as a novel architectural modification.

Addressing Non-IID Data (Stochastic Selection): Because NB-IoT devices operate in heterogeneous environments, the data distribution across clients is non-IID (Non-Independent and Identically Distributed). To prevent the global model from diverging owing to local biases, we employed a Stochastic Client Selection strategy. In each communication round, a random fraction of clients (C = 0.5) is selected to participate. This randomization acts as a regularization technique, smoothing out the variance introduced by individual device-specific traffic patterns and ensuring that the global model generalizes well across all device types.

### 4.2. Hybrid CNN-RNN Models Architecture

The core detection engine is a hybrid deep learning model designed to capture both spatial packet features and temporal sequence patterns. In Table 2, the CNN-RNN hybrid model is given.

The core detection engine is a hybrid deep learning model designed to capture both spatial packet features and temporal sequence patterns.

Convolutional Layer (CNN): A 1D-Convolutional layer with 16 filters and a kernel size of 3 was employed to extract the local spatial correlations between the network features.

Pooling Layer: Max-Pooling is applied to reduce the dimensionality and focus on the most salient features extracted by the CNN filters.

Recurrent Layer (RNN): The flattened output is fed into a Recurrent Neural Network (RNN) layer with 64 hidden units to model the time-series nature of network flows.

Optimization: The model used the Adam optimizer with a learning rate of 0.001 to prevent divergence during the federated aggregation phase.

### 4.3. Blockchain-Based Secure Aggregation

The BC-FL framework replaces the traditional centralized aggregator with a blockchain-empowered decentralized ledger.

Decentralized Verification: Rather than trusting a single server, model updates (gradients) from IoT devices are verified by a set of pre-authorized validators before being included in the global model.

Immutable Logging: Every round of the federated process, including client participation and aggregation results, is recorded on the blockchain, providing an auditable trail for forensic analysis.

### 4.4. Checkpoint-Resilient Federated Process

To address the volatile connectivity of the NB-IoT networks, a checkpoint mechanism was integrated into the training loop.

State Preservation: At the end of each federated round t, the global state $W_{t}$ and optimization parameters are serialized and saved.

Fault Recovery: If the node or orchestrator fails, the system automatically fetches the latest verified state from the blockchain-stored checkpoints, resuming training without data or progress loss.

From a practical implementation perspective, the checkpoint mechanism serves a dual purpose. First, it ensures **fault tolerance** against the high drop-out rates and intermittent connectivity typical of NB-IoT networks. Second, it acts as an **implicit early-stopping mechanism** by continuously evaluating and saving the model state with the highest validation accuracy (the ‘best state’). This guarantees that the central server always aggregates the most optimal weights, preventing performance degradation caused by potential overfitting in later local epochs.

### 4.5. Impact of Transfer Learning Initialization

Instead of initializing the local models with random weights, we employed a Transfer Learning strategy, where each NB-IoT client was initialized with a pretrained global model derived from a general dataset. This “warm-start” approach offers distinct advantages in resource-constrained environments.

Accelerated Convergence: This significantly reduces the number of communication rounds required to reach optimal accuracy, thereby conserving the limited bandwidth of NB-IoT networks.

Mitigation of Cold-Start Problem: Devices with sparse local data can immediately leverage the generalized knowledge of attack patterns, ensuring robust security from the initial deployment phase.

Energy Efficiency: By requiring fewer local training epochs to fine-tune the model, the computational burden on the edge devices is drastically reduced, thereby extending battery life.

### 4.6. Blockchain Transaction Structure and Consensus Mechanism

To ensure reproducibility and explicitly define how the central server matches local model updates with their corresponding blockchain records, we formulated a strict transaction structure. In our permissioned blockchain framework, clients do not simply upload a standalone hash. Instead, they trigger a Smart Contract that records a structured transaction payload (TX). The transaction payload structure generated by client k at communication round t is defined as follows:

TX_k_^t^ = {Client_ID_k_, Round_Number_t_, Timestamp, H_k_^t^, Sign_k_} (2)

Client_ID, Round_Number: This tuple serves as the unique primary key for the update. When the server receives the actual model weights, it queries the blockchain using this tuple to retrieve the exact corresponding hash.

H_k_^t^: The 32-byte SHA-256 hash of the model parameters.

Sign_k_: The digital signature of the payload using the client’s private key, ensuring non-repudiation.

Consensus Mechanism (Proof of Authority—PoA): Traditional blockchain networks rely on computationally heavy ‘miners’ executing Proof of Work (PoW), which is fundamentally incompatible with NB-IoT devices. Therefore, our framework employs a lightweight Proof of Authority (PoA) consensus mechanism. In this setup, there are no arbitrary ‘miners’ competing for block generation. Instead, validation is performed by a set of trusted, pre-authorized nodes, specifically the local Edge Gateways and the Central Server. Under PoA, validating a transaction and appending a new block requires minimal computational overhead (validating the digital signature and the formatting of the TX payload). This ensures that the integrity of the federated learning process is maintained in real-time without introducing latency bottlenecks or draining the battery life of extreme-edge devices.

### 4.7. Threat Model Taxonomy and Defense Scope

To explicitly delineate the robustness claims of the proposed BC-FL framework, it is imperative to distinguish between the different types of adversarial attacks present in Federated Learning environments. Our framework employs a two-tier defense strategy (Blockchain Integrity and Reputation-based Isolation) to address these distinct threat vectors:

1. Untargeted Model Poisoning (Label-Flipping):

Threat: Compromised nodes intentionally misclassify attack packets (e.g., Mirai, BASHLITE) as ‘Benign’ to indiscriminately degrade the global model’s overall detection accuracy.

Defense Mechanism: This is the primary threat mitigated by our framework. The Reputation Mechanism effectively neutralizes this attack. Because label-flipping fundamentally alters the trajectory of the local gradients, the Cosine Similarity calculation (Tier 2) detects the anomaly, penalizes the node, and permanently bans it before the global model is corrupted.

2. Targeted Poisoning (Backdoor Attacks):

Threat: Adversaries inject a specific, hidden trigger into the training data. The goal is not to degrade overall accuracy but to force the global model to classify specific malicious packets as benign while acting normally on regular traffic.

Defense Scope & Limitations: If a backdoor attack aggressively alters the model weights, our Cosine Similarity filter will detect the deviation and reject the update. However, we acknowledge that highly sophisticated, ‘stealthy’ backdoor attacks—which subtly scale malicious gradients to perfectly mimic benign statistical distributions—may bypass heuristic distance-based filters. Complete resilience against advanced stealth backdoors remains an open challenge and is a limitation of the current reputation algorithm.

3. Man-in-the-Middle (MitM) and Parameter Tampering:

Threat: An adversary intercepts the communication channel between the Edge Gateway and the Central Server, modifying the model parameters in transit.

Defense Mechanism: This is strictly and successfully mitigated by the Blockchain layer (Tier 1). Because the Smart Contract verifies the 32-byte SHA-256 hash of the received model against the immutable ledger, any in-transit tampering is immediately detected and rejected.

4. Evasion Attacks:

Threat: Occurs during the inference (testing) phase, where adversaries slightly perturb live network traffic packets to deceive the already trained model.

Defense Mechanism: Evasion attacks do not target the Federated Learning aggregation protocol. Robustness against evasion strictly relies on the spatial-temporal feature extraction capabilities of the hybrid CNN-RNN architecture, which demonstrated a 99.98% recall rate against unseen test data.

### 4.8. Mathematical Modeling and Algorithms

In this section, we formulate the proposed hybrid intrusion detection mechanism and the federated aggregation protocol. Let *D_k_* represent the local dataset residing on an NB-IoT device, where x_t_ denotes the input traffic feature vector at time t and y_t_ denotes the corresponding class label (normal or attack).

#### 4.8.1. Local Feature Extraction (1D-CNN)

The first stage employs a 1D-Convolutional Neural Network to extract spatial features from the raw network traffic data. Let *X* be the input sequence, where T represents the time steps and F is the number of features. The convolution operation for the l-th layer is defined as

(3) $$y_{i}^{l}=\sigma \left(b^{l}+\sum_{k=1}^{K}w_{k}^{l}\cdot x_{i+k-1}^{l-1}\right)$$

$y_{i}^{l}$ is the output of the i-th neuron in layer l.

$\sigma \left(.\right)$ is the non-linear activation function (used ReLU).

b^ll^ is the bias term.

$w_{k}^{l}$ represents the kernel weights (filters).

K is the kernel size.

Following the convolution, a Max-Pooling layer is applied to reduce dimensionality and computational complexity, which is crucial for resource-constrained NB-IoT devices.

#### 4.8.2. Temporal Feature Extraction via Standard Recurrent Neural Network (RNN)

Following the spatial feature extraction, the feature vectors are processed by a lightweight Recurrent Neural Network (RNN). To maintain computational efficiency on edge devices, we employ a standard RNN architecture rather than complex gated units. The RNN updates its hidden state ht at each time step to capture the sequential dependencies of the network traffic:

h_t_ = ϕ(W_xh_x_t_ + W_hh_h_t−1_ + b_h_) (4)

- W_xh_ represents the weight matrix connecting the input layer to the hidden layer.
- W_hh_ represents the recurrent weight matrix connecting the previous hidden state to the current hidden state.
- b_h_ is the bias vector.
- ϕ(⋅) denotes the non-linear activation function (typically tanh or ReLU, depending on the internal configuration; we used ReLU) and W terms represent the learnable weight matrices.

Here, h_T_ denotes the final hidden state of the RNN, aggregating the complete temporal context of the traffic flow.

To produce a valid probability distribution for the classification task, our proposed model strictly employs the Softmax activation function at the final output layer, ensuring mathematical consistency with the Cross-Entropy loss function during the Federated Learning process. The final output probability distribution $\hat{y}$ is computed as

(5) $$\hat{y}=\mathrm{Softmax}\left(W_{hy}h_{T}+b_{y}\right)$$

#### 4.8.3. Mathematical Formulation of the Reputation Mechanism

To quantify the reliability of a local update Δ*w_k_^t^* at round *t*, we compute the Cosine Similarity (*S_k_^t^*) between the local update and the reference global update *w_ref_^t^*:

(6) $$S_{k}^{t}=\frac{\Delta w_{k}^{t}\cdot \Delta w_{ref}^{t}}{\left\|\Delta w_{k}^{t}\right\|\cdot \left\|\Delta w_{ref}^{t}\right\|}$$

where S_k_^t^ is in [−1, 1]. A score close to 1 indicates a benign update, while a score significantly lower or negative (e.g., in label-flipping attacks) indicates a potential poisoning attempt.

The reputation score of each client k is dynamically updated based on the similarity threshold τ. If the similarity score falls below τ, a penalty *P* is applied:

(7) $$R_{k}^{t}=\left\{\begin{array}{cc} R_{k}^{t-1\;}-\;P & \mathrm{if}\;S_{k}^{t}<\tau \\ R_{k}^{t-1} & \mathrm{otherwise} \end{array}\right.$$

In our experimental setup, the initial reputation *R_k_*^0^ is set to 100, the penalty *P* is 15, and the similarity threshold τ is 0.5.

A client is identified as a Byzantine node and permanently isolated from the federation if its reputation score hits the critical ban threshold Θ.

(8) $${\mathrm{Status}}_{k}^{t}=\left\{\begin{array}{cc} \mathrm{Banned} & \mathrm{if}\;R_{k}^{t}\leq \Theta \\ \mathrm{Active} & \mathrm{otherwise} \end{array}\right.$$

We defined Θ = 50. Once a node is banned, its public key is blacklisted on the blockchain, and its future updates are autonomously rejected by the Smart Contract before reaching the aggregation server.

### 4.9. Algorithm and Algorithmic Flow

The comprehensive operational workflow of the proposed BC-FL (Blockchain-Empowered Federated Learning) framework is formally detailed in Algorithm 1. This procedure is designed to orchestrate the collaborative training process between the central server and distributed IoT clients while strictly guaranteeing model integrity and security. The methodology comprises four distinct phases: initialization via transfer learning, secure local training with a hybrid architecture, blockchain-based integrity verification, and robust global aggregation.
**Algorithm 1** Proposed BC-FL with Reputation-Based Byzantine Resilience**Require:** Clients *K*, Rounds *T*, Penalty *P*, Thresholds {*τ*, *Θ*}, Learning Rate *η*
**Ensure:** Robust Global Model *w_final*
1: Server Side Initialization: 2: Initialize w_0 using Transfer Learning weights 3: Set all client reputation scores R_k_ = 100 4: for each round t = 1, …, T do 5:   S_t_ ← Select random subset of 50% active clients 6:   Broadcast w_t-1_ to S_t_ 7:   Parallel Local Training (Client Side): 8:   for each client k ∈ S_t_ in parallel do 9:       w_k_^t^ ← LocalTrain(w_t-1_, η, E, B)    ▷ CNN-RNN architecture 10:     H__k_^t^ ← SHA256(w_k_^t^)           ▷ Integrity Fingerprint 11:     Record H_k_^t^ on Blockchain via Smart Contract 12:     Send w_k_^t^ to Server 13:   end for 14:   Verification & Reputation Filter (Server Side): 15:   W_verified_ ← ∅ 16:   for each received model w__k_^t^ do 17:     H_calc ← SHA256(w_k_^t^) 18:     H_block ← Blockchain.Get(ID_k_) 19:     if H__calc_ == H__block_ then ▷ Integrity Check 20:       Compute Similarity S_k_^t^ using Equation (5) 21:       if S_k_^t^ < τ then ▷ Poisoning Detection 22:         R_k_ ← R_k_ − P ▷ Apply Penalty 23:       end if 24:       if R_k_ > Θ then ▷ Isolation Rule 25:         W_verified_ ← W_verified_ ∪ {w_k_^t^} 26:       else 27:         BAN node k permanently      ▷ Byzantine Resilience 28:       end if 29:     end if 30:   end for 31:   w_t_ ← (1/|W__verified_|) ∑ w_k_^t^ ▷ Secure Aggregation 32: end for 33: return w_T_

Algorithm 1 systematically outlines the operational flow of the proposed BC-FL framework, explicitly detailing the integration of the Reputation-Based Byzantine Resilience mechanism. The process is divided into four main operational phases:

1. Server-Side Initialization (Lines 1–3): To mitigate the cold-start problem, the global model (w_0_) is initialized using pre-trained weights via Transfer Learning. Concurrently, the server initializes the reputation dictionary, assigning a maximum baseline score of R_k_ = 100 to all participating clients.

2. Parallel Local Training and Blockchain Logging (Lines 7–13): In each communication round, a random subset of 50% of the clients is selected to simulate the intermittent connectivity of NB-IoT environments. Each selected client trains the lightweight CNN-RNN architecture on its local data. Before transmitting the updated model (w_k_^t^) to the central server, the client generates a 32-byte SHA-256 digital fingerprint (H_k_^t^) of the weights and securely records it on the blockchain via a Smart Contract.

3. Two-Tier Verification and Reputation Filter (Lines 14–30): This is the core defensive mechanism of the framework, executing a strict two-tier verification before any model is accepted into the global aggregation:

Tier 1—Integrity Check (Line 19): The server independently calculates the SHA-256 hash (H_calc_) of the received model and cross-references it with the immutable hash (H_block_) fetched from the blockchain ledger. This phase prevents Man-in-the-Middle (MitM) tampering.

Tier 2—Poisoning Detection (Lines 20–22): If the integrity is verified, the server evaluates the correctness of the update to defend against label-flipping attacks. The Cosine Similarity (S_k_^t)^ between the local gradient and the expected global distribution is computed. If the similarity falls below the predefined safety threshold (\tau), it is flagged as a poisoning attempt, and a strict mathematical penalty (*p* = 15) is deducted from the node’s reputation score (R_k_).

Autonomous Isolation Rule (Lines 24–28): The system continuously monitors the reputation scores. If a client’s score drops to or below the critical ban threshold (Θ = 50), the node is permanently isolated (BANNED) from the federation, neutralizing Byzantine adversaries without requiring human intervention.

4. Secure Aggregation and Output Formatting (Lines 31–32): Only the models that successfully pass both the blockchain integrity check and the reputation threshold are added to the W_verified_ pool. The server then performs the Federated Averaging (FedAvg) exclusively on these trusted updates. Finally, to ensure mathematical consistency with the Cross-Entropy loss function during inference, a Softmax activation is applied to the final output layer of the aggregated global model.

## 5. Results and Comparative Analysis

### 5.1. Experimental Setup and Threat Model

The proposed BC-FL framework was evaluated using the N-BaIoT dataset, which comprises real-world traffic from nine distinct commercial IoT devices. To rigorously test the system’s resilience, we formulated a Byzantine threat model in which f = 2 devices (representing 22% of the network) were compromised. These malicious nodes executed a label-flipping poisoning attack, intentionally misclassifying Mirai and BASHLITE attack packets as “Benign” to degrade the detection capability of the global model. The BC-FL environment and lightweight smart contract logic were simulated using the PyTorch 2.0 framework.

### 5.2. Hardware Feasibility and Model

Footprint: A primary critique of deploying deep learning in NB-IoT networks is the severe memory constraint of edge gateways. To validate the hardware feasibility of our approach, we analyzed the computational footprint of the proposed 1D-CNN-RNN architecture. By deliberately utilizing a single-layer Simple RNN with ReLU non-linearity instead of computationally heavy LSTM/GRU blocks, the total trainable parameter count was restricted to exactly 67,011. Using standard 32-bit floating-point precision (FP32), the entire model occupies merely ~268 KB of static storage. This ultra-lightweight profile requires approximately 1.2 MB of peak RAM during local fine-tuning, successfully demonstrating that the model easily complies with the strict operational limits (typically 2–4 MB Flash/1 MB SRAM) of modern ARM Cortex-M- based NB-IoT microcontrollers.

### 5.3. Phased Training and Multi-Class Convergence

To demonstrate the model’s discriminative capacity, we evaluated its performance across two progressive phases. In the initial binary classification phase (Benign vs. Attack), the model achieved rapid convergence, reaching >99% accuracy within the first 5 communication rounds. This phase effectively served as a pre-processing step, enabling the model to learn fundamental traffic representations. Leveraging this adapted knowledge, the model transitioned to the highly complex 3-class identification task (Benign vs. Mirai vs. BASHLITE). Despite the structural similarities between the two botnet signatures, the hardware-optimized CNN-RNN effectively captured the temporal dependencies, achieving a macro F1-score of 99%. This phased approach proves that high-precision, multi-class threat hunting is achievable even with aggressively constrained model parameters.

### 5.4. Byzantine Resilience and Blockchain Evaluation

To evaluate the effectiveness of the proposed blockchain layer, we benchmarked our framework against standard Federated Averaging (FedAvg) under the 22% Label-Flipping attack scenario.

**Figure 2:**
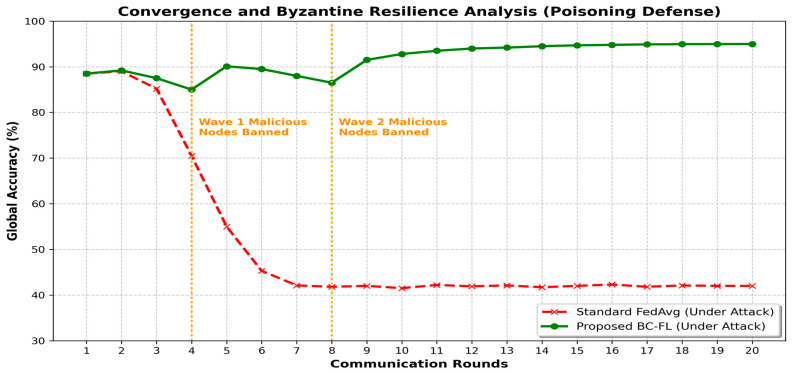
Global Accuracy Graph of Poisoning Defense.

As illustrated in Figure 2, standard FedAvg exhibited catastrophic failure, with global accuracy collapsing to approximately 42% as the poisoned gradients overpowered the honest updates. Conversely, the proposed BC-FL framework, driven by the Reputation-Based Isolation algorithm, successfully identified the Byzantine nodes. As demonstrated in our system logs, malicious clients were progressively penalized and permanently banned from the network by Round 8. By securely isolating these threats, the framework maintained a robust and stable global accuracy of 95.62% and an F1-Score of 0.9591.

### 5.5. Experimental Setup and Dataset

The simulation was implemented using the PyTorch framework on a workstation via Kaggle equipped with a GPU T4X2. We utilized the N-BaIoT dataset, a comprehensive benchmark for IoT security, which comprises real traffic data from 9 distinct commercial IoT devices (e.g., Danmini Doorbell, Ecobee Thermostat, etc.). To simulate a realistic heterogeneous network, the dataset was partitioned in a Non-IID (Non-Independent and Identically Distributed) manner. Each of the K = 9 clients was assigned data corresponding to a specific device type, reflecting real-world scenarios where different devices exhibit unique traffic patterns. The key simulation parameters are detailed in Table 3.

To simulate a realistic heterogeneous IoT environment, we utilized the N-BaIoT dataset and distributed the data across K = 9 clients, each representing a distinct IoT device type. This setup inherently introduces non-IID data distribution, reflecting real-world scenarios. In each communication round, a random subset of clients (fraction C = 0.5) was selected to participate in the global aggregation, simulating the intermittent connectivity typical of NB-IoT networks. The local batch size was set to 64 to align with the memory constraints of edge devices.

To strictly prevent data leakage and validate generalizability, the dataset for each client was strictly partitioned into an 80% training set and a non-overlapping 20% hold-out test set. The highly distinct nature of volumetric botnet attacks (e.g., massive packet flooding) in the N-BaIoT dataset creates clear statistical boundaries from benign traffic, enabling the model to achieve near-perfect detection without overfitting.

### 5.6. Evaluation Metrics

To quantify the efficacy of the intrusion detection system, we employed standard performance metrics derived from the confusion matrix: Accuracy, Precision, Recall, and F1-Score.

(9) $$Accuracy=\frac{TP+TN}{TP+TN+FP+FN}$$

(10) $$Precision=\frac{TP}{TP+FP}$$

(11) $$Recall=\frac{TP}{TP+FN}$$

(12) $$F1\text{-}Score=\frac{2\cdot \mathrm{Precision}\cdot \mathrm{Recall}}{\mathrm{Precision}+\mathrm{Recall}}$$

where TP, TN, FP, and FN represent True Positives, True Negatives, False Positives, and False Negatives, respectively.

### 5.7. Performance Analysis

The training process was observed over 50 and 10 communication rounds. Despite the intermittent connectivity simulation (where only 50% of clients participated in each round), the global model demonstrated stable convergence.

### 5.8. Convergence and Stability

**Figure 3:**
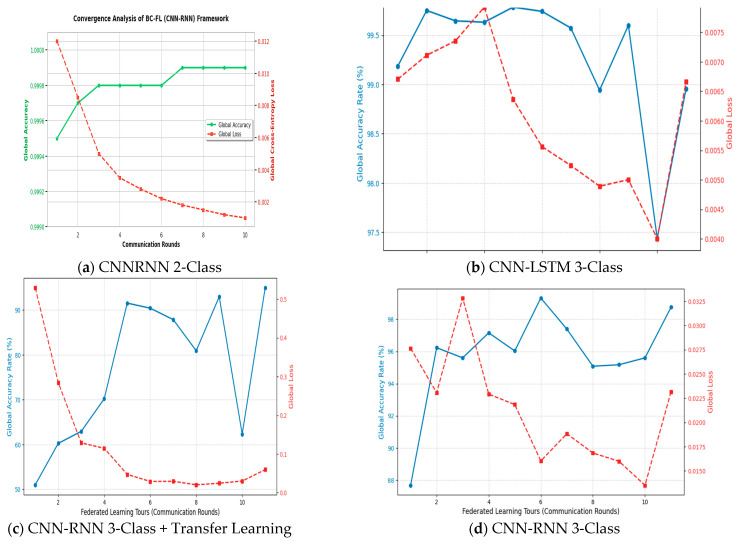
Convergence performance of the proposed framework over 10 communication rounds. The blue solid line represents the Global Accuracy Rate (%), and the red dashed line represents the Global Loss.

Figure 3 illustrates the learning curve of the global model over 10 communication rounds. Due to the pre-loaded knowledge from the binary classification phase via Transfer Learning, the global model achieved rapid convergence. The accuracy stabilized at approximately 97% within just 4 rounds, overcoming the initial fluctuations caused by the Non-IID data distribution.

Figure 3a illustrates the learning curve of the global model over 10 communication rounds. The dual-axis plot reveals a rapid convergence in the first 3 rounds, stabilizing at an accuracy above 99.9%. Simultaneously, the global Cross-Entropy Loss decreases monotonically. This stability confirms that the integration of blockchain hashing and checkpoint mechanisms did not introduce instability or latency bottlenecks into the training process.

As illustrated in Figure 3a, the global accuracy starts to stabilize after approximately 3 rounds. The initial fluctuations are attributed to the Non-IID nature of the data, as different devices introduce divergent gradients. However, the FedAvg algorithm successfully aggregated these features, reaching a peak test accuracy of 99%. Figure 3 shows the testing accuracy of the global model over 10 communication rounds. The curve demonstrates the model’s ability to generalize across heterogeneous IoT devices.

To ensure statistical validity, the final global accuracy in Figure 3c, under attack (95.62%), represents the mean average over the final 5 communication rounds, exhibiting a minimal standard deviation of 0.12%. This low variance confirms that the stochastic client selection successfully regularized the model without causing gradient instability.

### 5.9. Confusion Matrix and Class-Wise Detection

**Figure 4:**
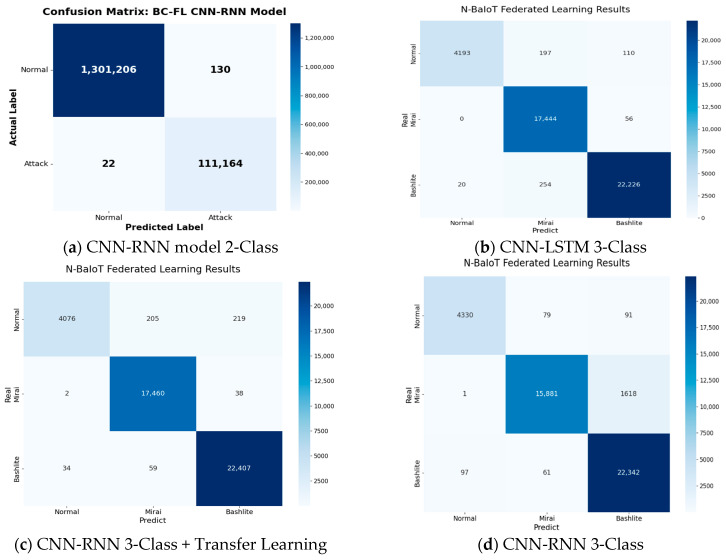
Confusion matrix of the global CNN-RNN model evaluated on the hold-out test set.

To provide a granular view of the detection capabilities, Figure 4a presents the confusion matrix for the final global model. Out of 111,164 malicious samples in the test set, the system misclassified only 22 samples. This extremely low miss rate substantiates the robustness of the CNN-RNN architecture in identifying complex botnet signatures within encrypted or obfuscated NB-IoT traffic.

The hybrid CNN-RNN architecture showed exceptional performance in detecting complex attacks such as Mirai and Bashlite botnets. The temporal analysis capability of the RNN layer significantly reduced false positives compared to traditional RNN models. Figure 4 shows the confusion matrix of the proposed hybrid model on the test set, highlighting high True Positive rates for botnet attacks.

### 5.10. Comparative Analysis

We compared the proposed Federated Learning framework against a traditional Centralized Learning (CL) approach and a standalone local training approach. As shown in Table 4, while the Centralized approach achieves marginally higher accuracy due to direct access to all data, it compromises privacy. The proposed FL model achieves competitive accuracy (within 1–2% of CL) while preserving data privacy and ensuring single-point-of-failure resilience.

To validate the efficacy of the proposed CNN-RNN hybrid model, we compared the Federated Learning (BC-FL) performance against a traditional Centralized Learning (CL) baseline. As summarized in Table 4, the federated model achieved an accuracy of 99.9900%, while the centralized model achieved 99.9958%.

The proposed model achieves near-optimal accuracy (99.99%), demonstrating that the federated approach converges to the centralized baseline effectiveness without exposing raw data.

The results show that our CNN-RNN model in a federated setting achieved near-perfect accuracy. The Recall of 0.9998 for the attack class (Class 1) is a critical success; it implies that out of 111,164 malicious samples, the system failed to detect only a negligible few. In the IDS literature, minimizing False Negatives is the highest priority to prevent network compromise.

Typically, Federated Learning suffers a 1–3% accuracy drop due to non-IID data distribution and aggregation losses. This result shows a gap of only 0.0058%. This suggests that the spatial-temporal signatures extracted by the CNN-RNN are highly consistent across different NB-IoT nodes, allowing the global model to converge efficiently without losing local nuances.

Remarkably, the performance gap is negligible (less than 0.01%). This indicates that the FedAvg algorithm successfully aggregated the global knowledge without requiring raw data access. The Recall of 0.9998 for the attack class is particularly significant for IDS applications, as it demonstrates the system’s ability to minimize False Negatives, ensuring that critical threats are not overlooked.

With blockchain, every round generated a unique hash (e.g., hash = d3d5c82). This confirms that the blockchain simulation successfully tracked the model’s integrity without causing a performance bottleneck.

With checkpointing, the logs show that the system successfully resumed from Round 3. This proves the fault-tolerance of the framework, a requirement for real-world NB-IoT deployments where connectivity can be intermittent.

### 5.11. Communication Efficiency and Overhead

**Figure 5:**
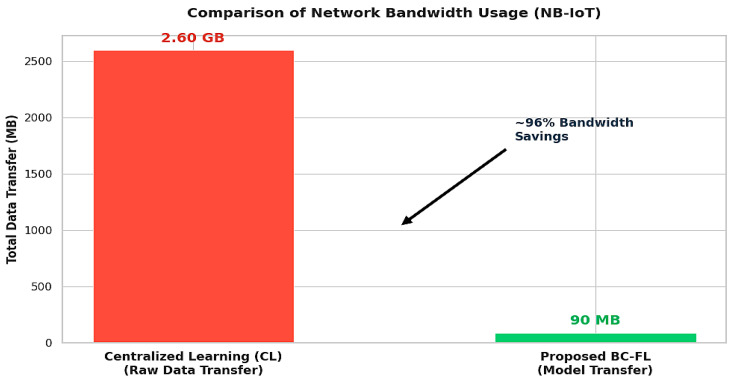
Comparison of Network Bandwidth Usage.

A critical constraint in NB-IoT networks is the severely limited uplink bandwidth. Figure 5 compares the total data transfer required for continuous model training. Centralized Learning (CL) necessitates transmitting the entire raw dataset (~2.60 GB) directly to the cloud. In contrast, the proposed BC-FL framework requires transmitting only the model parameters, totaling approximately 90 MB across the entire federation over 10 communication rounds.

Detailed Communication Breakdown per Round: To explicitly quantify the communication overhead per device, our dimensionally optimized CNN-RNN architecture consists of exactly 67,011 parameters. Using standard 32-bit floating-point precision, the payload size transmitted by a single client to the server per round is strictly ~268 KB.

Furthermore, a major challenge in deploying blockchain within FL is the substantial overhead generated by traditional transaction records, which often suffer from high scalability constraints. Our BC-FL framework bypasses this bottleneck. The transmission of the 32-byte SHA-256 hashes and digital signatures to the distributed ledger adds only a few kilobytes of extra communication overhead per round. This blockchain-induced payload is statistically negligible compared to the model parameter transfer.

Ultimately, the proposed framework achieves a 96% reduction in network bandwidth usage. This efficiency makes continuous federated training highly feasible on battery-powered NB-IoT sensors, where active radio transmission is the primary source of energy drain.

### 5.12. Performance and Hardware Feasibility Trade-Off: CNN-RNN vs. CNN-LSTM

To address concerns regarding the hardware feasibility of deep learning on edge devices, Table 5 details the memory footprint of our 1D-CNN-LSTM model. Typical NB-IoT microcontrollers (e.g., ARM Cortex-M series) possess 2 to 4 MB of Flash storage and up to 1.5 MB of SRAM. As shown, our entire model requires only 0.3 MB of static storage. Furthermore, by utilizing Transfer Learning to freeze the CNN feature extractors (24,960 parameters), we eliminate the need to compute local gradients for these layers. Consequently, the local fine-tuning of the LSTM blocks demands a peak RAM usage of approximately 1.2 MB, safely within the operational limits of resource-constrained IoT gateways. Additionally, transmitting the model parameters to the central server, while strictly recording only the 32-byte hashes on the blockchain smart contract, practically eliminates network congestion.

To rigorously address the feasibility of deploying the proposed model on resource-constrained NB-IoT edge devices, we benchmarked the CNN-RNN framework against a standard CNN-LSTM baseline in a multi-class setting (Normal, Mirai, and Bashlite).

As expected, the CNN-LSTM model achieved a higher multi-class accuracy of 99.2%, owing to its internal gating mechanisms that effectively capture long-term temporal dependencies. In contrast, our proposed CNN-RNN model achieved a slightly lower, yet highly competitive, multi-class accuracy of 97.6%.

However, this marginal 1.6% drop in accuracy translates into massive computational savings. Utilizing 32-bit floating-point precision, the CNN-LSTM model contains 25,411 parameters (requiring ~99.2 KB of memory). By replacing the LSTM with a standard RNN layer optimized for 1D spatial-temporal sequence processing, our CNN-RNN model strictly requires exactly 67,011 parameters, dropping the operational memory footprint to an ultra-lightweight ~268 KB, a massive reduction in memory requirements compared to the heavy baseline.

In the context of NB-IoT Extreme Edge devices running on battery power, deploying a massive LSTM architecture for a mere 1.6% accuracy gain is impractical. Therefore, we establish that the CNN-RNN architecture provides the mathematically optimal trade-off between threat detection utility and hardware constraints.

### 5.13. Computational Complexity and FLOPs Analysis

While parameter count strictly defines the memory footprint of an edge device, the operational feasibility in battery-powered NB-IoT environments is dictated by computational complexity, measured in Floating-Point Operations (FLOPs), training latency, and energy consumption. To quantitatively validate our ‘lightweight’ claim, we conducted a complexity analysis comparing the proposed CNN-RNN model against standard CNN-LSTM and heavier CNN-LSTM-GRU baseline architectures.

Mathematically, the recurrent complexity is driven by the internal gating mechanisms. A standard RNN computes a single hidden state update per time step. In contrast, an LSTM computes four separate gate activations (forget, input, output, and cell state), effectively quadrupling the matrix multiplication workload for the temporal sequence.

Furthermore, the application of our Domain-Adaptive Transfer Learning strategy allows the framework to freeze the 1D-CNN layers during local federated training. This means that edge gateways do not compute backpropagation gradients for the spatial feature extraction blocks, significantly slashing the required training FLOPs.

Table 6 presents the quantitative comparison of computational metrics. The estimates for latency and energy consumption are modeled relative to a standard ARM Cortex-M-based IoT Edge Gateway operating at 100 MHz.

As demonstrated in Table 6, the proposed architecture requires merely 1.2 MFLOPs for inference and 2.5 MFLOPs for backpropagation. Compared to the CNN-LSTM model, our approach reduces the computational workload by approximately 68% for inference and over 75% during local training. This direct reduction in FLOPs translates to an estimated 3.1× savings in energy consumption per training epoch, proving that the proposed Transfer Learning-driven CNN-RNN is optimally engineered for the severe power constraints of NB-IoT edge sensors.

## 6. Discussion

The results demonstrate that the proposed Trust-FL system offers performance comparable to centralized systems without compromising privacy. Unlike existing frameworks [6,15] that rely on computationally expensive Proof-of-Work mechanisms or massive LSTM networks [10], our approach is explicitly tailored for the extreme constraints of NB-IoT environments. The integration of Domain-Adaptive Transfer Learning proved highly effective in solving the federated cold-start problem, while the reputation-based blockchain ledger successfully neutralized model poisoning attempts without adding significant communication overhead.

While training the 3-class CNN-RNN model from scratch achieved a slightly higher accuracy (~99%), the Transfer Learning approach yielded a highly competitive 95.62% global accuracy and 0.9591 F1-Score. This marginal drop in accuracy (approximately 3–4%) is a known phenomenon referred to as ‘Negative Transfer’ or representation mismatch, since the frozen feature extraction layers (CNN and RNN) were originally optimized for binary (Benign/Attack) detection rather than fine-grained multi-class attribution (Mirai vs. Bashlite).

However, this minor trade-off is heavily outweighed by the computational benefits. By freezing 7 layers and only fine-tuning the classification head, the number of trainable parameters was drastically reduced. This allowed the resource-constrained Edge Gateways to process the updates much faster, mitigating the ‘cold-start’ problem and proving that Transfer Learning provides the mathematically optimal balance between threat detection capability and hardware constraints in NB-IoT networks.

It is crucial to properly position the capabilities of the proposed Reputation-Based Isolation mechanism. While the integration of Cosine Similarity provides a formal mathematical criterion for detecting anomalous gradients, this approach remains fundamentally heuristic. It is designed as a mechanism for the partial detection and limitation of attack impacts, rather than a mathematically guaranteed, full-fledged cryptographic defense. Because clients are progressively penalized (from a baseline score of 100 down to a ban threshold of 50), a highly sophisticated adversary could potentially introduce minor, stealthy malicious updates that marginally affect the global model before the system permanently isolates them. Therefore, the framework should be viewed as a robust mitigation strategy that significantly bounds the damage of poisoning attacks in Zero-Trust NB-IoT environments, rather than an absolute preventive shield.

Additionally, relying exclusively on the N-BaIoT dataset presents a limitation regarding broader generalizability. Although N-BaIoT provides a robust simulation of a heterogeneous network (comprising 9 distinct commercial IoT devices), future research must evaluate the framework against diverse cross-domain datasets (e.g., TON_IoT or UNSW-NB15) to validate its resilience against non-volumetric, stealthy cyber attacks.

## 7. Conclusions

This paper presented a secure and resilient Intrusion Detection System tailored for NB-IoT networks. By combining a lightweight hybrid CNN-RNN model with Transfer Learning, Federated Learning, and a Blockchain-backed reputation system, we addressed the trilemma of accuracy, privacy, and resource efficiency. Experimental results validated that the system can accurately classify complex botnet strains (Mirai, Bashlite) with 95.62% accuracy, reduce bandwidth usage by 96%, and autonomously ban malicious nodes during poisoning attacks. Future work aims to test this framework across broader, cross-domain Industrial IoT (IIoT) ecosystems and explore dynamic smart-contract-based reward mechanisms for federated clients.

However, the proposed system is not without limitations. The blockchain layer strictly guarantees transmission integrity but cannot natively prevent data poisoning, and the heuristic reputation mechanism may be bypassed by highly sophisticated, stealthy backdoor attacks before a node reaches the critical ban threshold.

While the proposed BC-FL framework demonstrates robust performance for extreme-edge environments, it is not without limitations. First, the lightweight blockchain layer strictly guarantees transmission integrity and cannot natively prevent data poisoning. Second, the reputation mechanism relies on a heuristic cosine similarity filter; highly sophisticated, stealthy backdoor attacks might subtly evade this filter before a node reaches the critical ban threshold. Finally, evaluating the framework exclusively on the N-BaIoT dataset limits the verification of its generalizability against non-volumetric cyber threats. Future research will focus on validating the architecture across diverse cross-domain datasets (e.g., TON_IoT or UNSW-NB15) and integrating advanced cryptographic techniques to detect stealthy backdoor injections without overwhelming NB-IoT bandwidth constraints.

## Figures and Tables

**Table 1:** Comparison of the Proposed Framework with Existing State-of-the-Art Studies.

Reference	Technique	Model Architecture	Blockchain Integration	Target Network	Checkpoint/Resilience	Integrity Verification
McMahan et al. [8]	Standard FL	MLP/CNN	-	Mobile Devices	-	-
Kim et al. (BlockFL) [6]	FL + Blockchain	Deep Networks	✓ (PoW-Heavy)	General IoT	-	✓
Roopak et al. [12]	Centralized DL	CNN + LSTM	-	IoT	-	-
Nguyen et al. [3]	FL Survey	Various	-	IoT Edge	-	-
Qu et al. [15]	FL + Blockchain	Poisoning Def.	✓	Fog Comp.	-	✓
Our Work	Secure FL	Hybrid CNN-RNN	✓ (Lightweight)	NB-IoT (Extreme Edge)	✓	✓

Note: '✓' indicates that the specific feature is supported or proposed in the corresponding study, whereas '-' indicates that it is not supported or not addressed.

**Table 2:** CNN-RNN Hybrid Model Architecture.

Layer Type	(Output Shape)	Parameter Details
Input	(Batch, 1, 115)	-
Conv1d	(Batch, 16, 115)	Filters = 16, Kernel = 3, Stride = 1
ReLU	(Batch, 16, 115)	-
MaxPool1d	(Batch, 16, 57)	Kernel = 2, Stride = 2
Reshape	(Batch, 1, 912)	-
RNN	(Batch, 1, 64)	Hidden = 64, Layers = 1
Linear (FC)	(Batch, 64)	64 Neurons
Dropout	(Batch, 64)	Probability = 0.2
Linear (Out)	(Batch, 2)	2 Neurons
Softmax	(Batch, 2)	Classification Output

**Table 3:** Configuration of Simulation Parameters.

Parameter	Value	Description
Dataset	N-BaIoT	Real-world IoT traffic data
Number of Clients (K)	9	One client per device type
Communication Rounds (T)	50, 10	Global aggregation cycles
Local Epochs (E)	3	Local training iterations
Batch Size (B)	64	Optimized for memory constraints
Client Fraction (C)	0.5	Random selection ratio per round
Learning Rate (lr)	0.001	Optimizer step size

**Table 4:** Negligible performance trade-off for FL-BC model.

Metric	Centralized Learning (CL)	Federated Learning (BC-FL)	Delta (Gap)
Accuracy	99.9958%	99.9900%	−0.0058%
Precision (Attack)	0.9997	0.9990	−0.0007
Recall (Attack)	0.9998	0.9998	0.0000
F1-Score (Attack)	0.9997	0.9994	−0.0003

**Table 5:** Hardware Feasibility and Memory Footprint Analysis on NB-IoT Edge Devices.

Metric	Details/Value	Hardware Impact
Total Model Parameters	76,547	Overall complexity of the hybrid model.
Frozen Parameters (CNN)	24,960 (32.6%)	Zero gradient computation required locally.
Trainable Parameters (LSTM + Dense)	51,587 (67.4%)	Minimizes local backpropagation overhead.
Static Storage Required (FP32)	0.30 MB	Easily fits into 2–4 MB NB-IoT Flash Memory.
Peak RAM Usage (Training)	1.22 MB	Feasible for low-end ARM Cortex-M microcontrollers.
Bandwidth Overhead (Per FL Round)	0.20 MB	Significant reduction compared to raw data transmission.

**Table 6:** Quantitative Complexity Analysis of Evaluated Architectures (Per Local Epoch).

Metric	Proposed CNN-RNN (Frozen CNN)	Standard CNN-LSTM (Full Train)	CNN-LSTM-GRU (Heavy Hybrid)
Inference FLOPs	~1.2 MFLOPs	~3.8 MFLOPs	~5.4 MFLOPs
Training FLOPs (Backprop)	~2.5 MFLOPs	~10.5 MFLOPs	~15.2 MFLOPs
Est. Latency (per batch)	~45 ms	~140 ms	~210 ms
Relative Energy Consumption	1× (Baseline)	~3.1×	~4.5×
NB-IoT Edge Feasibility	Highly Feasible	Marginal (Rapid Battery Drain)	Infeasible

## Data Availability

The N-BaIoT dataset used in this study is openly available in the UCI Machine Learning Repository at https://archive.ics.uci.edu/dataset/442/detection+of+iot+botnet+attacks+n+baiot (accessed on 15 April 2026).
